# A template to quantify the location and density of CD3 + and CD8 + tumor-infiltrating lymphocytes in colon cancer by digital pathology on whole slides for an objective, standardized immune score assessment

**DOI:** 10.1007/s00262-020-02834-y

**Published:** 2021-01-13

**Authors:** Dordi Lea, Martin Watson, Ivar Skaland, Hanne R. Hagland, Melinda Lillesand, Einar Gudlaugsson, Kjetil Søreide

**Affiliations:** 1 Gastrointestinal Translational Research Unit, Molecular Laboratory, Hillevåg, Stavanger University Hospital, Stavanger, Norway; 2 Department of Clinical Medicine, University of Bergen, Bergen, Norway; 3 Department of Pathology, Stavanger University Hospital, Stavanger, Norway; 4 Department of Chemistry, Bioscience and Environmental Engineering, Faculty of Science and Technology, University of Stavanger, Stavanger, Norway; 5 Department of Gastrointestinal Surgery, Stavanger University Hospital, Stavanger, Norway

**Keywords:** Colorectal cancer, Immune response, Tumor-infiltrating lymphocytes, Tumor center, Tumor-invasive margin, Digital image analysis

## Abstract

**Background:**

In colon cancer, the location and density of tumor-infiltrating lymphocytes (TILs) can classify patients into low and high-risk groups for prognostication. While a commercially available ‘Immunoscore^®^’ exists, the incurred expenses and copyrights may prevent universal use. The aim of this study was to develop a robust and objective quantification method of TILs in colon cancer.

**Methods:**

A consecutive, unselected series of specimens from patients with colon cancer were available for immunohistochemistry and assessment of TILs by automated digital pathology. CD3 + and CD8 + cells at the invasive margin and in tumor center were assessed on consecutive sections using automated digital pathology and image analysis software (Visiopharm^®^). An algorithm template for whole slide assessment, generated cell counts per square millimeters (cells/mm^2^), from which the immune score was calculated using distribution volumes. Furthermore, immune score was compared with clinical and histopathological characteristics to confirm its relevance.

**Results:**

Based on the quantified TILs numbers by digital image analyses, patients were classified into low (*n* = 83, 69.7%), intermediate (*n* = 14, 11.8%) and high (*n* = 22, 18.5%) immune score groups. High immune score was associated with stage I–II tumors (*p* = 0.017) and a higher prevalence of microsatellite instable (MSI) tumors (*p* = 0.030). MSI tumors had a significantly higher numbers of CD3 + TILs in the invasive margin and CD8 + TILs in both tumor center and invasive margin, compared to microsatellite stable (MSS) tumors.

**Conclusion:**

A digital template to quantify an easy-to-use immune score corresponds with clinicopathological features and MSI in colon cancer.

**Supplementary Information:**

The online version contains supplementary material available at 10.1007/s00262-020-02834-y.

## Introduction

Colorectal cancer (CRC) is a leading cause to the cancer burden and cancer deaths worldwide. Despite improvements in surgical and oncological management over the last decade [1], about half of all patients will develop metastasis and eventually die from disseminated disease [2]. The Tumor–node–metastases system (TNM classification) used for staging and prognostication is imperfect in its ability to correctly guide treatment and define appropriate subgroups beyond surgical treatment [3]. The TNM system largely dependent on using the node status to guide further adjuvant treatment, and as a consequence there is ongoing risk for under- and overtreatment of patients, based on the current guidelines for adjuvant chemotherapy [4, 5].

Of note, emerging data suggest the role of molecular subtypes with distinct features and associated outcomes [6, 7]. Among the suggested consensus molecular subtypes is the “immunogenic” type, which is associated with hypermutation, microsatellite instability (MSI) and a favorable prognosis. Abundant immune cells are found in the vicinity of such tumors and the type, density and location of the immune cells within tumor samples strongly influence the evolution of CRCs [8, 9] with impact on prognosis reported in large, multicenter studies [10–12]. This adaptive immune response of T-cells in tumor has been quantified as a measure called “Immunoscore^®^” (HalioDx, Marseille, France) [11, 13], and is available commercially as a test [14]. However, the costs implied with the commercially available assay may be prohibitive in a public health care setting and/or may currently not be reimbursed for clinical routine use. More widespread use of immune scoring could be available if easy, accessible and low-cost methods would allow for stratification of immunogenic tumors. Moreover, manual and subjective assessment such as counting cells, is increasingly being replaced by digital pathology in routine practice in departments of pathology [15, 16]. The benefits of digital pathology include objective measurement on regular slides [17] with a quantitative read of how many cells of interest are present in an area using immunostained sample slides. The highly objectivity and quantitative approach makes it easier to compare high number of tissue slides from patients and correlate to disease outcome.

The aim of this study was to establish an objective and highly reproducible quantification method for tumor-infiltrating lymphocytes (TILs) in colon cancer and to correlate immune score to clinicopathological characteristics and MSI status.

## Methods

### Study design

Patients were recruited from an ongoing prospective, clinical-molecular biomarker outcomes study, the ACROBATICC project [18] (clinicaltrials.gov ID: NCT01762813). This cohort study is reported according to the STROBE [19] and the REMARK [20] guidelines for biomarker studies.

### Compliance with ethical standards

The study is conducted in accordance to national regulations and approved by the Norwegian Regional Ethics Committee (REK Helse Vest, #2012/742). All procedures performed in studies involving human participants were in accordance with the ethical standards of the institutional and/or national research committee and with the 1964 Helsinki Declaration and its later amendments or comparable ethical standards.

### Informed consent

Written informed consent was obtained from all participants prior to inclusion in the study.

### Study population

All patients were diagnosed, managed and followed-up at Stavanger University Hospital (SUH), a public-funded university hospital within the universal health care system of Norway. The protocol [18] and study cohort have been described in further detail elsewhere [21, 22]. The current study is based on patients with stage I–III colon cancer from the initial cohort recruited between January 2013 and May 2014 [21] that did not undergo neoadjuvant treatment. Of 132 consecutive stage I–III colon cancers, 119 were included in the study. Patients with two or more invasive colon carcinomas at time of surgery were excluded from the study, as these tumors might have a different biology [23]. When multiple tumor blocks were present, the tumor block that included invasive margin and most immune cells was selected for analysis [24].

### Histopathology

All specimens were staged (AJCC 8th edition) [25, 26] by board certified pathologists using a standardized gross pathology and microscopic histopathology template for reporting.

### Immunohistochemistry

Antigen retrieval and antibody dilution were optimized prior to the study onset. Adjacent to the hematoxylin–eosin (H&E) stained sections, consecutive 2 µm paraffin sections were cut and mounted onto Superfrost Plus slides (Menzel, Braunschweig, Germany), along the principles suggest previously [27]. The CD3 and CD8 slides were incubated at 60 °C for 1 h and then placed in the autostainer (Dako Omnis), where they were subject to an automated protocol as per manufacturer instructions, with a pretreatment at 97 °C in 30 min. CD3 (Dako Clone F7.2.38) was diluted with Dako Antibody diluent by 1:75 and CD8 (Dako Clone C8/144B) by 1:50. A peroxidase detection kit (Envision substrate working solution, Dako, Glostrup, Denmark) visualized the immune complex for all the antibodies. Sections were then counterstained with hematoxylin in the Dako Omnis stainer. Afterwards, the slides were dehydrated and mounted manually.

### Digital pathology assessment

**Fig. 1:**
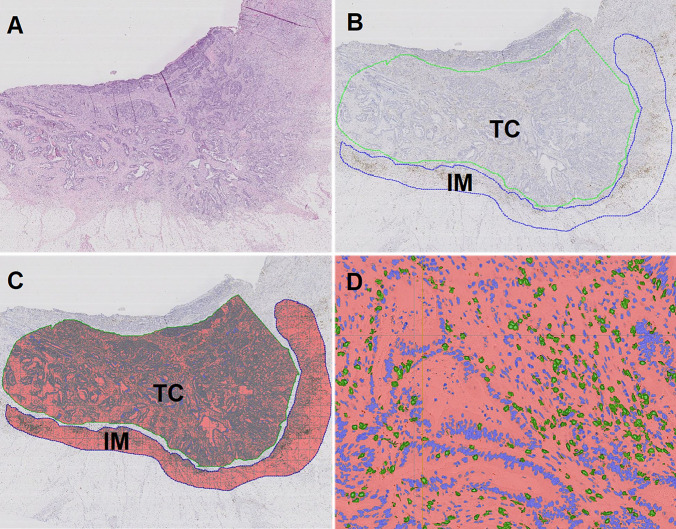
**a** Hematoxylin–eosin staining of tumor. **b** Immunohistochemical staining of CD3 (brown) in the same tumor with marking of tumor center/TC (blue) and invasive margin/IM (green). **c** Digital image analysis measured the CD3 + tumor-infiltrating lymphocytes (TILs) in TC and IM. The number of positive TILs was calculated pr. mm^2^. The same tumor area was analyzed for CD3 and CD8 for each patient. **d** Close-up view that shows positive TILs marked with green. Negative nuclei are marked blue and surrounding stroma is marked red

CD3- and CD8-stained slides were scanned at 40 × magnification using Leica SCN400 slide scanner (Leica Microsystems, Wetzlar, Germany) and uploaded to image analysis software, Visiopharm^®^ (Hoersholm, Denmark). The region of tumor center (TC) and invasive margin (IM) were marked manually on whole slides in Visiopharm^®^ and these regions (region of interest, ROI) were used for CD3 + and CD8 + cell quantification (Fig. 1). Visiopharm^®^ identified and measured the area of positive cells (µm) using digital image analysis (DIA). After DIA, the area of positive cells were transformed into number of positive TILs based on the estimation of mean area of a lymphocyte (60 µm^2^). The number of positive CD3 + and CD8^+^ TILs was calculated per square millimeters (n cells/mm^2^) [28], further represented as density of cells.

Unspecified stains and artefacts were removed manually using the image software. Application in the image software was adjusted for detection of different immune stain intensity.

**Fig. 2:**
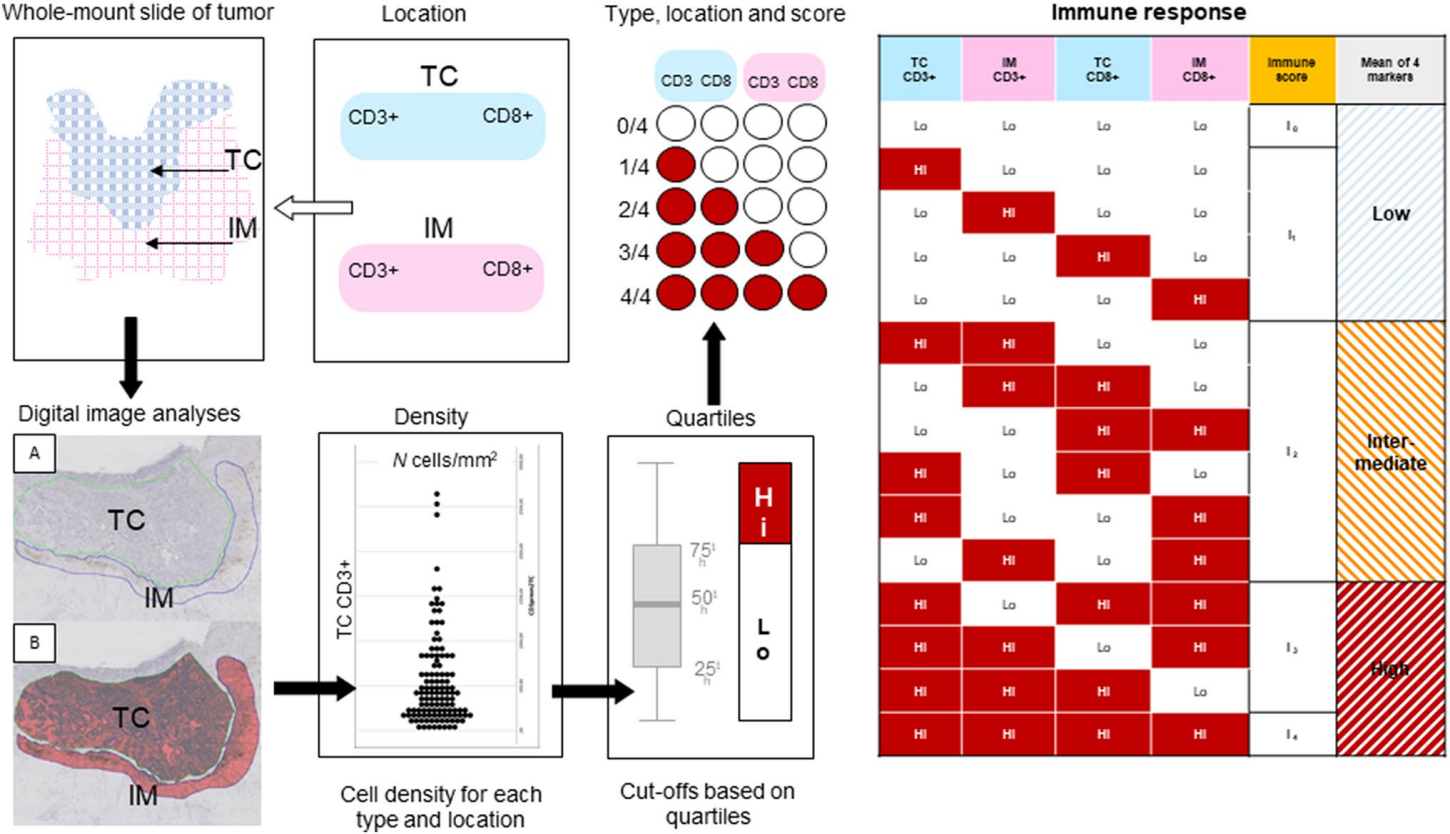
Flowchart for calculating immune response based on mean densities of CD3 + and CD8 + in tumor center (TC) and invasive margin (IM)

Immune response was calculated based on mean densities of CD3 + and CD8 + in TC and IM in all of the patients in the study. The calculated mean density was used to divide the individual cases into “high” or “low” immune response. Cases with mean density ≥ 75-percentile were regarded as “high” immune response. Patients were stratified from I0 to I4 according to the “Immunoscore^®^”, based on the total number of observed high densities (CD3^+^ TILs and CD8^+^ TILs in TC and IM) [11, 13]. The final immune score was categorized based on mean percentiles for all four markers, and divided into immune score “low”, “intermediate” and “high” based on the number of markers (0–4) ≥ 75th percentile (Fig. 2).

### Histopathological parameters

Histopathological parameters were registered from the pathology report including mucinous component, lymphovascular infiltration and lymph node status. In addition, tumor budding was registered in the HE section with deepest infiltration according to recommended guidelines [29, 30].

### Analysis of microsatellite instability

Analysis of MSI has been described previously [31, 32]. Briefly, FFPE blocks were selected by an experienced pathologist (DL) and 4 × 10 μm sections were cut at a microtome. Automated DNA extraction was carried out using AllPrep DNA/RNA FFPE kit (Qiagen, Hilden, Germany) on a QiaCUBE instrument (Qiagen) according to manufacturer’s instructions. Nucleic acid concentration and purity were measured on a NanoDrop 2000 (ThermoFischer scientific, Waltham, USA). Multiplex PCR reactions (one for each MSI) were set up for tumor and normal DNA in each patient. TypeIT microsatellite (Qiagen) master mix, together with a blending of 5 × 5′-fluorescently labelled primer pairs was used for each reaction. PCR conditions were as follows: 5′ at 95 °C (initial denaturation and enzyme activation), followed by 37 cycles of 30″ at 95 °C (denaturation), 90″ at 55 (MSI) and 30″ at 72 °C (extension). A final extension step for 30′ at 60 °C. The primers for MSI were specific for BAT-25, BAT-26, NR-21, NR-24 and NR-27 [33, 34], which are all quasimonomorphic mononucleotide repeats with a high fidelity to high-frequency MSI, as shown previously [32]. To define a tumor as MSI, at least 2/5 markers needed to be unstable in their panels.

### Statistics

IBM SPSS Statistics for Windows, Version 26.0 (IBM Corporation, Armonk, NY, USA) was used for statistical analysis. Associations between categorical variables were tested with Chi-square. Mann–Whitney *U* test was used to compare differences in continuous or ordinal variables between groups. All tests were two-tailed and a *p* value < 0.050 was determined as statistically significant.

## Results

The study cohort included 119 stage I–III colon cancer patients that underwent surgery with curative intent. Patient characteristics is presented in Supplement Table 1. According to the TNM classification, the distribution between stage I–III were approximately equal (31%, 36% and 32%, respectively). Slightly more women were noted in the cohort, otherwise the distributions were as expected for a consecutive cohort of colon cancer, with lymph node status, tumor size, histological type and grade, and overall age.

**Fig. 3:**
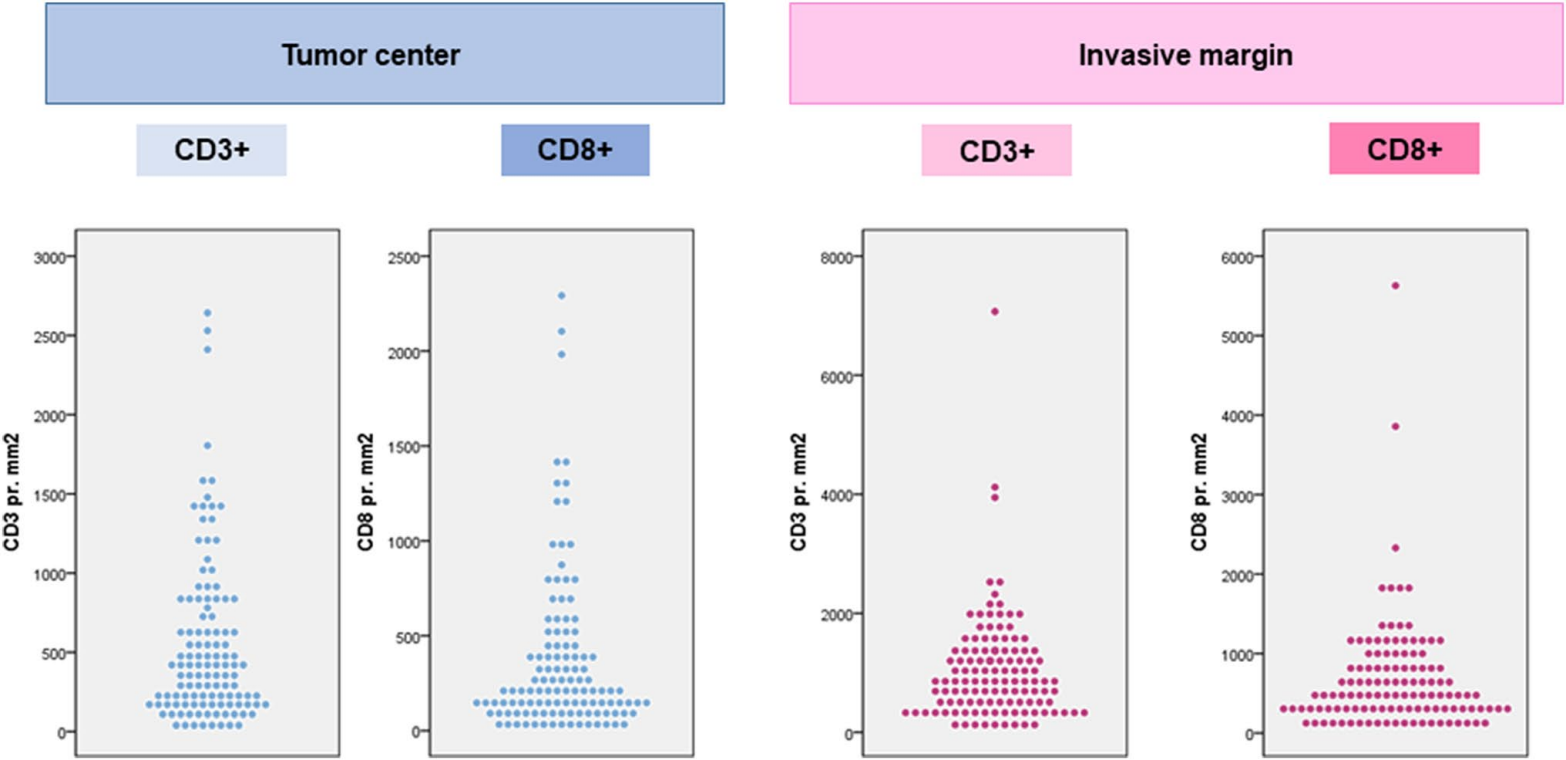
Distribution of number of CD3 + and CD8 + TILs in tumor center (TC) and invasive margin (IM)

Distribution of the number of CD3 + and CD8 + TILs in TC and IM is presented in Fig. 3. The number of TILs was higher in IM compared with TC, both for CD3 + and CD8 + cells. Percentiles for evaluating immune response based on density (cells/mm^2^) of CD3 and CD8 is presented in Table 1. The total numbers of cells/mm^2^ counted in the upper range (75th percentile) were almost double in the invasive margin compared to tumor center, for both CD3 + and CD8 + cells, respectively. Table 2 shows distribution of cases with high immune response (≥ 75th percentile) in the different regions. These results were summarized to calculate immune score, which is presented in Table 3. According to the immune score set up (Table 3), there number of patients in the immune score groups of low, intermediate and high was 83 (69.7%), 14 (11.8%) and 22 (18.5%), respectively. Hence, two-thirds of the colon cancers were deemed immune-low, with the immune-high cases split even between a three of four and four of four regions marked as immune high.

**Table 1:** Density (cells/mm^2^) cut-off values based on highest quartiles (75th percentile)

		Tumor center		Invasive margin	
		CD3 +	CD8 +	CD3 +	CD8 +
		cells/mm^2^	cells/mm^2^	cells/mm^2^	cells/mm^2^
Median		393	220	858	513
Percentiles	25th	187	112	452	277
	50th	393	220	858	513
	75th	760	466	1390	896

**Table 2:** Distribution of patients (*n*) with high immune response (≥ 75th percentile) in different regions

	Tumor center				Invasive margin			
	CD3 Low	CD3 High	CD8 Low	CD8 High	CD3 Low	CD3 High	CD8 Low	CD8 High
0 of 4	69	0	69	0	69	0	69	0
1 of 4	13	1	12	2	9	5	8	6
2 of 4	5	9	5	9	8	6	10	4
3 of 4	3	8	3	8	3	8	2	9
4 of 4	0	11	0	11	0	11	0	11
Total	90	29	89	30	89	30	89	30

**Table 3:** Immune score based on high tumor density of CD3 and CD8 in different regions

Number of regions	Patients (*n*)	Means of markers
0 of 4	69	Low
1 of 4	14	
2 of 4	14	Intermediate
3 of 4	11	High
4 of 4	11	
Total	119	

Higher immune scores were associated with a higher frequency of stage I–II tumors (*p* = 0.017) and a higher prevalence of MSI tumors (*p* = 0.030), compared with tumors from intermediate and low immune score. Three of 22 patients with high immune score had stage III disease, whereas for stage I and II the number was 12 and 7, respectively. For patients with low immune score, 19 were stage I, 34 stage II and 30 stage III. For intermediate immune score, the corresponding number of patients were 6 stage I, 2 stage II and 6 stage III. Twelve of 42 MSI tumors (28.6%) had high immune score and 10 of 77 microsatellite stabile (MSS) tumors (13.0%) had a high immune score, responding to twofold increase of immune-high cases in the MSI colon cancers.

**Fig. 4:**
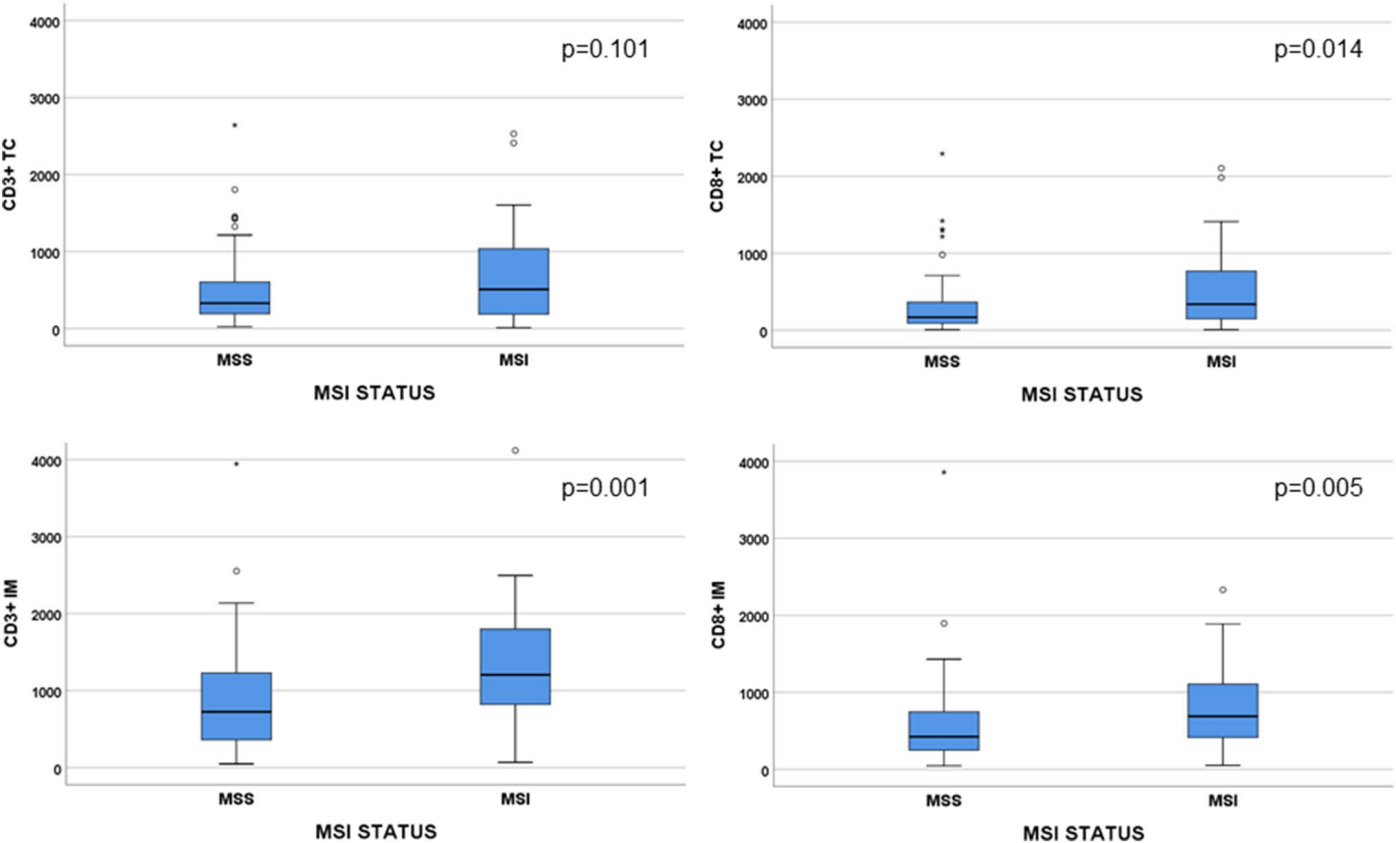
Comparison of number of CD3 + and CD8 + tumor-infiltrating lymphocytes (TILs) of MSS and MSI tumors in tumor center (TC) and invasive margin (IM). Extreme values > 4000 cells is not shown in figure

Tumors with MSI had a significantly higher number of CD3 + TILs in IM and CD8 + TILs in both TC and IM (Fig. 4). There was no significant association between high immune score and sex, median age, localization, grade, tumor size, N-status, tumor budding, lymphovascular or perineural infiltration.

## Discussion

In this study, we present an objective automated, digital quantification method of CD3 + and CD8 + lymphocytes at IM and in TC in colon cancer. The resultant immune score is strongly associated with TNM stage and microsatellite instability, two well-documented and very strong prognostic factors in colon cancer. The immune score allowed to stratify patients into low, intermediate and high immune response groups. The quantification should be easy to use by other digital pathology laboratories and represent a robust and objective approach to immune cell quantification in colon cancer specimens.

The method is based on the construct principle of the “Immunoscore”, which has been validated in a large international cohort series [11]. However, this commercially available Immunoscore^®^ (HalioDx, Marseille, France) is adapted to certain manufactures of antibodies and autostainer [11], and may prevents laboratories from setting up the method with available equipment in the laboratory. Widespread evaluation and dissemination may thus be hampered. Hence, the current quantification may represent an alternative measurement that is adoptable, easy to implement, affordable and objective, yet provides transparency for reproduction.

We found that the number of TILs is significantly higher at the IM than in TC, corresponding to a previously reported study [35]. Furthermore, our study showed that patients with high immune score were associated with an earlier stage of the disease, which might explain why high immune score is associated with better prognosis. Several studies show that TILs play a significant role for prognosis in colon cancer. Mlecnik et al. found that a high density of CD8 + TILs is associated with reduced risk of relapse [35], whereas Angell and co-workers [36] found that tumors with reduced numbers of CD8 + had a higher risk of metastasis. A recent meta-analysis of 22 studies including 5108 patients by Zhao and co-workers, found that high CD3 + infiltrates in colon cancer correlated with improved cancer-specific survival and overall survival. Furthermore, the same study found that high density of CD3 + in IM indicated increased disease-free survival (DFS) and high CD8 + in TC was associated with improved DFS [37]. The abovementioned studies all support that scoring TILs in colon cancer can give valuable prognostic information.

In the NICHE study [38], the investigators explored the safety and efficiency of neoadjuvant immunotherapy (ipilimumab and nivolumab) in operable colon cancers. Despite being a small phase I/II study with over half being MSI cancer, a remarkable response was found (pathological response in 20/20 MSI patients; 19 had major pathological response and 12 had complete pathological response. Even in tumors with MSS, 4 of 15 had response [38]. As density of immune cells were related to response, a pre-treatment biopsy may become important. While not investigated in the NICHE study, the current template for immune score by digital pathology may become essential to select the patients who would benefit from such treatment in the future. Of note, half the patients in the NICHE study had MSI tumors, suggesting a selection of the included patients. The current rate of 35% MSI is high, but reflect that only colon (and no rectal) cancers were selected for the cohort and, is in line with previously reported data for such cohorts [39].

So far, and to the best of our knowledge, no biomarkers based on digital image analysis (DIA), has been used in pathological classification of colon cancer [24], despite digital pathology now being steadily introduced in routine diagnostic practice at several pathology departments. While there is a lack of consensus biomarkers for use, the implementation and spread in use makes it easier to use DIA in diagnostic setting and to perform prognostic scoring. For immune score to become an international recognized standard, it is important that it is available through affordable software and that the method is transparent. Others have applied deep learning methods to analyze TILs in HE sections [40, 41] and have found association with survival.

The added time and cost to stain and score relies on a couple of assumptions. One, a digital platform needs to be in place. We recognize that not all pathology labs may have this readily available, but increasingly this is being rolled out as the way forward to standardize scoring in quantitative pathology. Second, the time taken for a technician or bioengineer to cut slides and prepare counts from the template is time efficient. If standardized and introduced into the routine pathway of clinical work, the estimated extra time for a bioengineer to cut, stain and prep for digital analysis would be in the range of 15–20 min; the pathologist’s time to mark the area for digital analyses would be part of the routine clinical work and add a maximum of 30 min, but probably less. Hence, the use of this score should not be labor intensive nor require extensive human hours of labor.

The cost implied (given that digital pathology instruments are available in a given pathology lab) amounts to reagents for immunohistochemistry markers. These are usually available already in most labs, and in general inexpensive (estimated at around 10 Euros per slide), but with variable costs between countries. Taken together, we believe that the template for an automated immune score presented here would be both time efficient and cost containing.

Our study has some limitations to address, with one being the size of the cohort. A larger cohort size might have demonstrated more differences between the clinicopathological parameters in the patients to the high immune score versus the patients with intermediate and low immune score. Furthermore, the DIA method is not validated against manual pathology evaluation using a microscope and counting cells. This is near impossible (from a time and labour perspective implied) due to the high number of positive cells found in each tissue slide. However, the less time-consuming and labour-efficient results obtained by DIA exemplifies the strength of using automated digital pathology to this extent. The international effort of validating the “Immunoscore^®^” as a prognostic marker demonstrated value of the commercial test [11]. Future studies using easy-to-use, available, objective and reproducible methods to assess TILs in colon cancer may facilitate its wider dissemination and clinical implementation. With further validation, internally and externally, the role of the current template-based immune score should arrive at clinically relevant use and be able to designate appropriate subgroups of patients stratified to their relevant therapy decisions.

## Conclusion

A whole slide, digital pathology template using imaging software was developed to quantify immune score. Known clinicopathological features like MSI status correlated with a higher immune infiltrate, exemplified by a greater immune score. Large-scale internal and external validation to demonstrate robustness and generalizability for clinical use is ongoing.

### Supplementary Information

Below is the link to the electronic supplementary material.

Supplementary file1 (DOCX 18 KB)

## References

[1] Nedrebø BS, Søreide K, Eriksen MT, Dørum LM, Kvaloy JT, Soreide JA (2011). Survival effect of implementing national treatment strategies for curatively resected colonic and rectal cancer. Br J Surg.

[2] Safiri S, Sepanlou SG, Ikuta KS, Bisignano C, Salimzadeh H, Delavari A, Ansari R, Roshandel G, Merat S, Fitzmaurice C, Force LM (2019). The global, regional, and national burden of colorectal cancer and its attributable risk factors in 195 countries and territories, 1990–2017: a systematic analysis for the Global Burden of Disease Study 2017. Lancet Gastroenterol Hepatol.

[3] Lea D, Håland S, Hagland HR, Søreide K (2014). Accuracy of TNM staging in colorectal cancer: a review of current culprits, the modern role of morphology and stepping-stones for improvements in the molecular era. Scand J Gastroenterol.

[4] Nagtegaal ID, Quirke P, Schmoll HJ (2012). Has the new TNM classification for colorectal cancer improved care?. Nat Rev Clin Oncol.

[5] Puppa G, Sonzogni A, Colombari R, Pelosi G (2010). TNM staging system of colorectal carcinoma: a critical appraisal of challenging issues. Arch Pathol Lab Med.

[6] Dienstmann R, Vermeulen L, Guinney J, Kopetz S, Tejpar S, Tabernero J (2017). Consensus molecular subtypes and the evolution of precision medicine in colorectal cancer. Nat Rev Cancer.

[7] Wang W, Kandimalla R, Huang H, Zhu L, Li Y, Gao F (2019). Molecular subtyping of colorectal cancer: recent progress, new challenges and emerging opportunities. Semin Cancer Biol.

[8] Galon J, Costes A, Sanchez-Cabo F, Kirilovsky A, Mlecnik B, Lagorce-Pages C (2006). Type, density, and location of immune cells within human colorectal tumors predict clinical outcome. Science.

[9] Pages F, Berger A, Camus M, Sanchez-Cabo F, Costes A, Molidor R (2005). Effector memory T cells, early metastasis, and survival in colorectal cancer. N Engl J Med.

[10] Ferris RL, Galon J (2016). Additional Support for the Introduction of Immune Cell Quantification in Colorectal Cancer Classification. J Natl Cancer Inst.

[11] Pagès F, Mlecnik B, Marliot F, Bindea G, Ou FS, Bifulco C (2018). International validation of the consensus Immunoscore for the classification of colon cancer: a prognostic and accuracy study. Lancet.

[12] Sun G, Dong X, Tang X, Qu H, Zhang H, Zhao E (2019). The prognostic value of immunoscore in patients with colorectal cancer: a systematic review and meta-analysis. Cancer Med.

[13] Galon J, Mlecnik B, Bindea G, Angell HK, Berger A, Lagorce C (2014). Towards the introduction of the 'Immunoscore' in the classification of malignant tumours. J Pathol.

[14] Blair HA (2020). Immunoscore(®): a diagnostic assay for clinical management of colon cancer. Mol Diagn Ther.

[15] Thorstenson S, Molin J, Lundstrom C (2014). Implementation of large-scale routine diagnostics using whole slide imaging in Sweden: digital pathology experiences 2006–2013. J Pathol Inf.

[16] Cheng CL, Azhar R, Sng SH, Chua YQ, Hwang JS, Chin JP (2016). Enabling digital pathology in the diagnostic setting: navigating through the implementation journey in an academic medical centre. J Clin Pathol.

[17] Veta M, van Diest PJ, Willems SM, Wang H, Madabhushi A, Cruz-Roa A (2015). Assessment of algorithms for mitosis detection in breast cancer histopathology images. Med Image Anal.

[18] Søreide K, Watson MM, Lea D, Nordgård O, Søreide JA, Hagland HR (2016). Assessment of clinically related outcomes and biomarker analysis for translational integration in colorectal cancer (ACROBATICC): study protocol for a population-based, consecutive cohort of surgically treated colorectal cancers and resected colorectal liver metastasis. J Transl Med.

[19] Ev E, Altman DG, Egger M, Pocock SJ, Gøtzsche PC, Vandenbroucke JP (2007). Strengthening the Reporting of Observational Studies in Epidemiology (STROBE) statement: guidelines for reporting observational studies. BMJ (Clin Res ed.).

[20] McShane LM, Altman DG, Sauerbrei W, Taube SE, Gion M, Clark GM (2005). REporting recommendations for tumour MARKer prognostic studies (REMARK). Br J Cancer.

[21] Watson MM, Kanani A, Lea D, Khajavi RB, Søreide JA, Kørner H (2020). Elevated microsatellite alterations at selected tetranucleotides (EMAST) in colorectal cancer is associated with an elderly, frail phenotype and improved recurrence-free survival. Ann Surg Oncol.

[22] Watson MM, Lea D, Gudlaugsson E, Skaland I, Hagland HR, Søreide K (2020). Prevalence of PD-L1 expression is associated with EMAST, density of peritumoral T-cells and recurrence-free survival in operable non-metastatic colorectal cancer. Cancer Immunol Immunother.

[23] Koness RJ, King TC, Schechter S, McLean SF, Lodowsky C, Wanebo HJ (1996). Synchronous colon carcinomas: molecular-genetic evidence for multicentricity. Ann Surg Oncol.

[24] Angell HK, Bruni D, Barrett JC, Herbst R, Galon J (2020). The immunoscore: colon cancer and beyond. Clin Cancer Res.

[25] AJCC Cancer Staging Manual. 8 ed. Springer International Publishing AG, Switzerland; 2017

[26] WHO Classification of Tumours, Digestive System Tumours. 5 ed. IARC Publications; 2019

[27] Hermitte F (2016). Biomarkers immune monitoring technology primer: Immunoscore(R) Colon. J Immunother Cancer.

[28] Hagland HR, Lea D, Watson MM, Soreide K (2017). Correlation of blood T-cells to intratumoural density and location of CD3+ and CD8+ T-cells in colorectal cancer. Anticancer Res.

[29] Cho SJ, Kakar S (2018). Tumor budding in colorectal carcinoma: translating a morphologic score into clinically meaningful results. Arch Pathol Lab Med.

[30] Lugli A, Kirsch R, Ajioka Y, Bosman F, Cathomas G, Dawson H (2017). Recommendations for reporting tumor budding in colorectal cancer based on the International Tumor Budding Consensus Conference (ITBCC) 2016. Modern Pathol Off J USA Can Acad Pathol.

[31] Watson MM, Kanani A, Lea D, Khajavi RB, Søreide JA, Kørner H (2019). Elevated microsatellite alterations at selected tetranucleotides (EMAST) in colorectal cancer is associated with an elderly, frail phenotype and improved recurrence-free survival. Ann Surg Oncol.

[32] Soreide K (2011). High-fidelity of five quasimonomorphic mononucleotide repeats to high-frequency microsatellite instability distribution in early-stage adenocarcinoma of the colon. Anticancer Res.

[33] Buhard O, Suraweera N, Lectard A, Duval A, Hamelin R (2004). Quasimonomorphic mononucleotide repeats for high-level microsatellite instability analysis. Dis Markers.

[34] Suraweera N, Duval A, Reperant M, Vaury C, Furlan D, Leroy K (2002). Evaluation of tumor microsatellite instability using five quasimonomorphic mononucleotide repeats and pentaplex PCR. Gastroenterology.

[35] Mlecnik B, Tosolini M, Kirilovsky A, Berger A, Bindea G, Meatchi T (2011). Histopathologic-based prognostic factors of colorectal cancers are associated with the state of the local immune reaction. J Clin Oncol.

[36] Angell HK, Gray N, Womack C, Pritchard DI, Wilkinson RW, Cumberbatch M (2013). Digital pattern recognition-based image analysis quantifies immune infiltrates in distinct tissue regions of colorectal cancer and identifies a metastatic phenotype. Br J Cancer.

[37] Zhao Y, Ge X, He J, Cheng Y, Wang Z, Wang J (2019). The prognostic value of tumor-infiltrating lymphocytes in colorectal cancer differs by anatomical subsite: a systematic review and meta-analysis. World J Surg Oncol.

[38] Chalabi M, Fanchi LF, Dijkstra KK, Van den Berg JG, Aalbers AG, Sikorska K (2020). Neoadjuvant immunotherapy leads to pathological responses in MMR-proficient and MMR-deficient early-stage colon cancers. Nat Med.

[39] Guastadisegni C, Colafranceschi M, Ottini L, Dogliotti E (2010). Microsatellite instability as a marker of prognosis and response to therapy: a meta-analysis of colorectal cancer survival data. Eur J Cancer.

[40] Saltz J, Gupta R, Hou L, Kurc T, Singh P, Nguyen V (2018). Spatial organization and molecular correlation of tumor-infiltrating lymphocytes using deep learning on pathology images. Cell Rep.

[41] Skrede OJ, De Raedt S, Kleppe A, Hveem TS, Liestol K, Maddison J (2020). Deep learning for prediction of colorectal cancer outcome: a discovery and validation study. Lancet (London, England).

