# Supplementary material for: A template to quantify the location and density of CD3 + and CD8 + tumor-infiltrating lymphocytes in colon cancer by digital pathology on whole slides for an objective, standardized immune score assessment
Source: Cancer Immunol Immunother. 2021 Jan 13;70(7):2049–57. doi: 10.1007/s00262-020-02834-y (PMC8195795; doi:10.1007/s00262-020-02834-y)
Supplement: Supplementary file 1 — Supplementary file1 (DOCX 18 KB) [file 262_2020_2834_MOESM1_ESM.docx]

**Table 1. Patient characteristics**

| **Characteristics** | **Variable/Cut-off** | **Total N, (%)** |
| --- | --- | --- |
| **Age (years)** | ≤73  >73 | 56 (47.1)  63 (52.9) |
| **Sex** | Male | 48 (40.3) |
|  | Female | 71 (59.7) |
| **Within Colon** | Proximal | 68 (57.1) |
|  | Distal | 51 (42.9) |
| **TNM-Stage** | I | 37 (31.1) |
|  | II | 43 (36.1) |
|  | III | 39 (32.8) |
| **Lymph node status** | N0 | 80 (67.2) |
|  | N+ | 39 (32.8) |
| **Tumor size (mm)** | ≤45mm  >45mm | 61 (51.2)  57 (48.3) |
| **Histological type** | Adenocarcinoma | 102 (85.7) |
|  | Mucinous carcinoma | 17 (14.3) |
| **Grade** | Low grade  High grade | 82 (68.9)  37 (31.1) |
| **Perineural invasion** | Present  Absent | 9 (7.6)  119 (92.4) |
| **Lymphovascular invasion** | Present  Absent | 43 (36.1)  76 (63.9) |
| **Tumor budding** | Low (<5)  Intermediate (5-9)  High (>9) | 58 (53.2)  28 (25.7)  23 (21.1) |
| **MSI-status** | MSS MSI | 77 (64.7)  42 (35.3) |

Age and tumor size is divided into two groups based on median value.

Tumor budding: 10 cases could not be assessed.

MSI: Microsatellite instable. MSS: Microsatellite stable
